# Relationship Between HIV Coinfection, Interleukin 10 Production, and *Mycobacterium tuberculosis* in Human Lymph Node Granulomas

**DOI:** 10.1093/infdis/jiw313

**Published:** 2016-07-26

**Authors:** Collin R. Diedrich, Jennifer O'Hern, Maximiliano G. Gutierrez, Nafiesa Allie, Patricia Papier, Graeme Meintjes, Anna K. Coussens, Helen Wainwright, Robert J. Wilkinson

**Affiliations:** 1 Clinical Infectious Diseases Research Initiative, Institute of Infectious Disease and Molecular Medicine; 2 Division of Anatomical Pathology; 3 Department of Medicine, University of Cape Town, South Africa; 4 Francis Crick Institute Mill Hill Laboratory; 5 Department of Medicine, Imperial College London, United Kingdom

**Keywords:** HIV, tuberculosis, granuloma, mycobacterium, coinfection, histology, lymph node, antiretroviral therapy, IL-10

## Abstract

***Background.*** Human immunodeficiency virus type 1 (HIV)–infected persons are more susceptible to tuberculosis than HIV–uninfected persons. Low peripheral CD4^+^ T-cell count is not the sole cause of higher susceptibility, because HIV–infected persons with a high peripheral CD4^+^ T-cell count and those prescribed successful antiretroviral therapy (ART) remain more prone to active tuberculosis than HIV–uninfected persons. We hypothesized that the increase in susceptibility is caused by the ability of HIV to manipulate *Mycobacterium tuberculosis*–associated granulomas.

***Methods.*** We examined 71 excised cervical lymph nodes (LNs) from persons with HIV and *M. tuberculosis* coinfection, those with HIV monoinfection, and those with *M. tuberculosis* monoinfection with a spectrum of peripheral CD4^+^ T-cell counts and ART statuses. We quantified differences in *M. tuberculosis* levels, HIV p24 levels, cellular response, and cytokine presence within granulomas.

***Results.*** HIV increased *M. tuberculosis* numbers and reduced CD4^+^ T-cell counts within granulomas. Peripheral CD4^+^ T-cell depletion correlated with granulomas that contained fewer CD4^+^ and CD8^+^ T cells, less interferon γ, more neutrophils, more interleukin 10 (IL-10), and increased *M. tuberculosis* numbers. *M. tuberculosis* numbers correlated positively with IL-10 and interferon α levels and fewer CD4^+^ and CD8^+^ T cells. ART reduced IL-10 production.

***Conclusions.*** Peripheral CD4^+^ T-cell depletion correlated with increased *M. tuberculosis* presence, increased IL-10 production, and other phenotypic changes within granulomas, demonstrating the HIV infection progressively changes these granulomas.

**(See the editorial commentary by Canaday and Toossi on pages 1292–3.)**

Human immunodeficiency virus type 1 (HIV) infection increases tuberculosis susceptibility [1] to such a great extent that tuberculosis is the leading cause of death of HIV–infected persons [2]. In 2013, an estimated 1.1 million new cases of tuberculosis and more than half (360 000) of all tuberculosis-associated deaths (500 000) occurred in coinfected persons [1]. These HIV-associated deaths and high incidence rates of tuberculosis occur despite the availability of antiretroviral therapy (ART) and tuberculosis preventive therapy. This demonstrates a need to understand better how HIV exacerbates tuberculosis immunopathology.

The reduction of the number of peripheral CD4^+^ T cells associated with HIV progression correlates with increased tuberculosis susceptibility. However a considerable increase in susceptibility occurs even prior to substantial peripheral CD4^+^ T-cell depletion and remains high in persons receiving antiretroviral therapy (ART) with no detectable viremia [3], suggesting that depletion of peripheral CD4^+^ T-cell counts and high viral load are not the only factors involved in increasing tuberculosis susceptibility. HIV–mediated destruction of CD4^+^ T cells is important to tuberculosis pathology because these cells are necessary for the appropriate response to *M. tuberculosis* [4, 5]. HIV also leads to a reduction in ex vivo peripheral [6, 7] and airway [8, 9] T-cell cytokine production following *M. tuberculosis* antigen stimulation. HIV infection has also been shown in vitro to increase the growth of *M. tuberculosis* in coinfected macrophages [10, 11], but such responses are highly variable [12]. Although these studies demonstrate that HIV causes specific reductions in the immunological responses to *M. tuberculosis*, very few studies have clearly demonstrated how HIV changes these responses within granulomas [13], the archetypal microscopic hallmark of tuberculosis-diseased tissue. The immunological function and cellular composition of granulomas significantly varies between granulomas [14] and blood or airways [15], demonstrating the need to examine granulomas directly [16].

*M. tuberculosis* granulomas function as organized immunological barriers that can prevent *M. tuberculosis* dissemination and growth or act as a reservoir for *M. tuberculosis* growth [14, 17]. *M. tuberculosis* granulomas are highly dynamic and organized and are active immunological responses to *M. tuberculosis*, with a homeostatic balance of necessary and equally important proinflammatory and antiinflammatory responses [14, 18]. Immunological disruptions within this delicate balance, such as CD4^+^ T-cell [5] and tumor necrosis factor (TNF) neutralization [19], can lead to increased tuberculosis pathology.

Granulomas can form in any tissue but are predominately found in the lungs and lymph nodes (LNs) of coinfected persons [1]. LNs are the primary site for the development of adaptive immunity, and they facilitate the dissemination of extrapulmonary tuberculosis [20], a form of disease that is more frequent in HIV–infected persons [1]. LNs are also primary sites for HIV infection, and their chronic infection by HIV results in significant CD4^+^ T-cell death, LN dysregulation, and T-cell exhaustion [21]. Understanding the interaction between HIV and *M. tuberculosis* at this diseased site may help elucidate how HIV increases *M. tuberculosis* dissemination and tuberculosis susceptibility.

Studies that have examined granulomas in persons with HIV and *M. tuberculosis* coinfection (hereafter, “coninfected persons”) and those with *M. tuberculosis* monoinfection are highly variable, and it is difficult to draw firm conclusions about how HIV changes granuloma organization, formation, cellular composition, and *M. tuberculosis* abundance [13]. The inconsistency of these data partially results from the highly variable and heterogeneous nature of granulomas, a limited number of studies, a low number of persons in the studies, and highly variable and qualitative methods, which make comparisons among multiple studies difficult. Moreover, determining whether HIV causes a disruption in the delicate immunological balance within a granuloma is important in understanding the mechanistic cause of the ability of HIV to increase tuberculosis susceptibility [22].

To address the limited data available focusing on *M. tuberculosis* granulomas within coinfected persons, we used an unbiased blinded technique to analyze granulomas from excised cervical LNs from both coinfected persons (with a spectrum of peripheral CD4^+^ T-cell counts and ART statuses) and *M. tuberculosis*–monoinfected persons. Whole-slide imaging of hematoxylin and eosin, acid-fast bacilli (AFB), and chromogenic staining for HIV p24, CD3, CD4, CD8, CD15, CD68, interferon γ (IFN-γ), IFN-α, TNF, and IL-10 were used to visualize phenotypic differences within all granulomas and the entire LN section. We determined quantitatively and qualitatively how granulomas not only differ between coinfected persons and *M. tuberculosis*–monoinfected persons, but also how HIV disease progression (characterized by a reduction in the peripheral CD4^+^ T-cell count) and ART phenotypically changed the site of tuberculosis diseased lymph nodes.

## METHODS

### Study Approval

This study was performed using excised cervical LN tissue stored within the Department of Anatomical Pathology at Groote Schuur Hospital (Cape Town, South Africa). All biopsy specimens were collected for clinical indications. Residual paraffin-embedded blocks of these specimens were stored for further processing. This study complied with the declaration of Helsinki (2008), and ethics approval was obtained from the University of Cape Town Human Research Ethics Committee (REC187/2013). Informed consent was waived, as this was a retrospective study of FFPE tissue samples collected during the course of routine clinical practice. Patient identifiers were unavailable to investigators.

### Patient Selection

The study group comprised persons who had undergone cervical LN biopsy between 2008 and 2013 and subsequently received a diagnosis of tuberculous lymphadenitis or an HIV diagnosis (for the HIV-mono-infected control group). Clinical case notes and pathology results were reviewed for demographic, clinical, pathologic, and microbiologic details for each individual identified. Patients were included if they had a diagnosis of tuberculosis lymphadenitis either via microbiological confirmation or if histologic findings and clinical picture were both consistent with the diagnosis. *M. tuberculosis* was directly identified within LNs by culture (based on detection of colony-forming units [CFU]), acid-fast bacilli (AFB) staining, or polymerase chain reaction (PCR) in 14 of 15 *M. tuberculosis*–monoinfected patients, 28 of 29 coinfected patients who were not receiving ART, and 8 of 11 coinfected patients receiving ART. The MGIT-BACTEC system was used to determine colony-forming unit positivity and approximate bacterial load by identifying how many days were needed for positivity [23]. For the 5 patients in this study without microbiological confirmation, the tuberculosis diagnosis was inferred from a positive sputum culture (for 1 patient) or on the basis of histologic findings consistent with tuberculosis (necrotizing and caseous granulomas; for 4 patients). These patients also had other factors supportive of their tuberculosis diagnosis: infiltrate observed on radiography, a mass enlarging over multiple months, ongoing lymphadenopathy and pleural effusion, and successful response to tuberculosis treatment after biopsy.

Patients also had known HIV status; for those with positive test result, a known peripheral CD4^+^ T-cell count was necessary. Of the HIV-positive patients without tuberculosis, the indication for biopsy was cervical lymphadenopathy; histologic analysis demonstrated reactive change only or no abnormality in 14 of 16 cases, and in 2 cases the LN was collected incidentally as part of surgery for trauma or other indication, with no pathology reported on histologic analysis.

### LN Processing

Cervical LN biopsy specimens were fixed in formalin within 1–2 hours after collection. They were then processed and embedded in paraffin wax, using typical protocols (formalin-fixed paraffin-embedded [FFPE] blocks). FFPE tissue blocks were serially sectioned at 4–6-mm thickness.

### Immunohistochemistry

The first section from each patient was stained with hematoxylin and eosin (CS701, CS700; Dako, Carpinteria, California), using product instructions. AFB were identified using Ziehl-Neelsen stain (; TB Color modified 100 497; Merck-Millipore, Darmstadt, Germany). The following antibodies were used to stain cells and cytokines: polyclonal rabbit anti-human CD3 (Dako; 1:300 dilution), monoclonal rabbit anti-human CD4 (SP35 clone, dilution 1:50; Cell Marque, Rocklin, California), monoclonal rabbit anti-human CD8 (SP16, 1:50; Cell Marque), mouse monoclonal anti-human CD68 (KP1, 1:1000; Dako), monoclonal mouse anti-human CD15 (LeuM1, 1:50; Abcam), monoclonal mouse anti-human HIVp24 (Kal-1, 1:20; Dako), polyclonal rabbit anti-human IFN-γ (1:150; Abcam, Cambridge, Massachusetts), monoclonal mouse anti-human TNF (52B83, 1:125; Abcam), polyclonal rabbit anti-human IL-10 (1:2000; Abcam), and polyclonal rabbit anti-human IFN-α (1:100; antibodies-online, Atlanta, GA). Appropriate isotypes were used to confirm staining specificity.

Immunohistochemistry followed standard protocols. Sections were deparaffinized in xylene and hydrated in graded EtOH and water. Two antigen-retrieval methods were used for appropriate antibodies: (1) CD3-, CD4-, CD8-, CD68-, and TNF-stained slides used ethylenediaminetetraacetic acid (EDTA) buffer (1 mM EDTA, 0.05% Tween 20, pH 8.0) at 100°C in a pressure cooker for 1.5 minutes; and (2) CD15-, IFN-α–, IL-10–, and IFN-γ–stained slides used citrate-EDTA buffer (10 mM citric acid, 2 mM EDTA, 0.05% Tween 20, pH 6.2) at 95°C–100°C for 20 minutes. After slides cooled, endogenous peroxidases were blocked with 2% H­_2_0_2_ in phosphate-buffered saline (PBS; 20 minutes). Antigen sites were blocked using a 1:20 dilution of goat serum in PBS for 2 hours at room temperature. Slides were stained for 12 hours at 4°C. Either horseradish peroxidase–conjugated polyclonal goat anti-mouse or anti-rabbit (Dako) secondary antibodies were used for 20 minutes at room temperature. Chromogenic DAB staining followed for 3 minutes (Liquid DAB+; Dako), followed by incubation with copper sulfate for 5 minutes (Sigma-Aldrich, St Louis, MO) and a hematoxylin counterstain. Slides were then dehydrated and mounted. Olympus VS120 Scanning Microscope (Olympus, Tokyo, Japan) with a 20× objective semiautomatically captured the entire slide area.

### Analysis

LN area, granuloma counts, granuloma size(s), granuloma type (caseous, unorganized inflammation, and solid cellular), LN architecture, AFB positivity, and HIV p24 positivity were all manually quantified over the entire tissue section, using Cellsens visualization software (Olympus, Tokyo, Japan). To quantify cellular and cytokine stains, the entire LN image was converted into a series of consecutive 100-MB TIFF images. To reduce bias, each TIFF image was analyzed with unique Cell Profiler (Broad Institute) pipelines to automatically quantify DAB-positive staining by quantifying cell counts (for CD3^+^, CD4^+^, and CD8^+^ T cells) that colocalized with nuclei or the total area stained (for CD15, CD68, TNF, IL-10, IFN-γ, and IFN-α) divided by the total LN section area. The total DAB area stained was quantified because necrotic areas that contained CD15 and CD68 staining were more diffuse and antibodies often stained areas without nuclei.

Individual CellProfiler pipelines were created for each antibody used with DAB staining. Each TIFF image was resized by a factor 0.5 to conserve processing power. Individual colors (DAB and hematoxylin) were then unmixed. Identification of primary objects (DAB or hematoxylin) was determined by pixel units, global threshold strategy, and Otsu thresholding methods. DAB coverage was determined for all markers, and colocalization of nuclei and DAB staining in CD3^+^, CD4^+^, and CD8^+^ T cells was quantified. The accuracy of each pipeline was confirmed with manual quantification.

### Statistical Analysis

Nonparametric Kruskal–Wallis, Mann–Whitney, or χ^2^ tests (with significance set at *P* < .05) were used to compare multiple groups or just 2 groups, respectively. Linear regression analysis was used to determine correlations in staining coverage. All statistics were conducted in GraphPad Prism v6.0 (La Jolla, California).

## RESULTS

### Patient Groups

Patients were organized into 4 groups: HIV monoinfected (n = 16; median age, 34 years [range, 23–70 years]; 56% female), *M. tuberculosis* monoinfected (n = 15; median age, 30 years [range, 18–68 years]; 53% female), coinfected without ART (n = 29; median age, 30 years [range, 16–48 years]; 68% female), and coinfected with ART (n = 11; 29 years [age, 25–48 years]; 62% female). Median peripheral CD4^+^ T-cell counts were obtained for all HIV infected persons, with values of 331 cells/μL of blood (range, 84–647 cells/μL of blood) in the HIV–monoinfected group, 145 cells/μL of blood (range, 11–591 cells/μL of blood) in the coinfected group without ART, and 204 cells/μL of blood (range, 29–347 cells/μL of blood) in the coinfected group with ART.

### Gross Pathology of Granulomas

**Figure 1.**
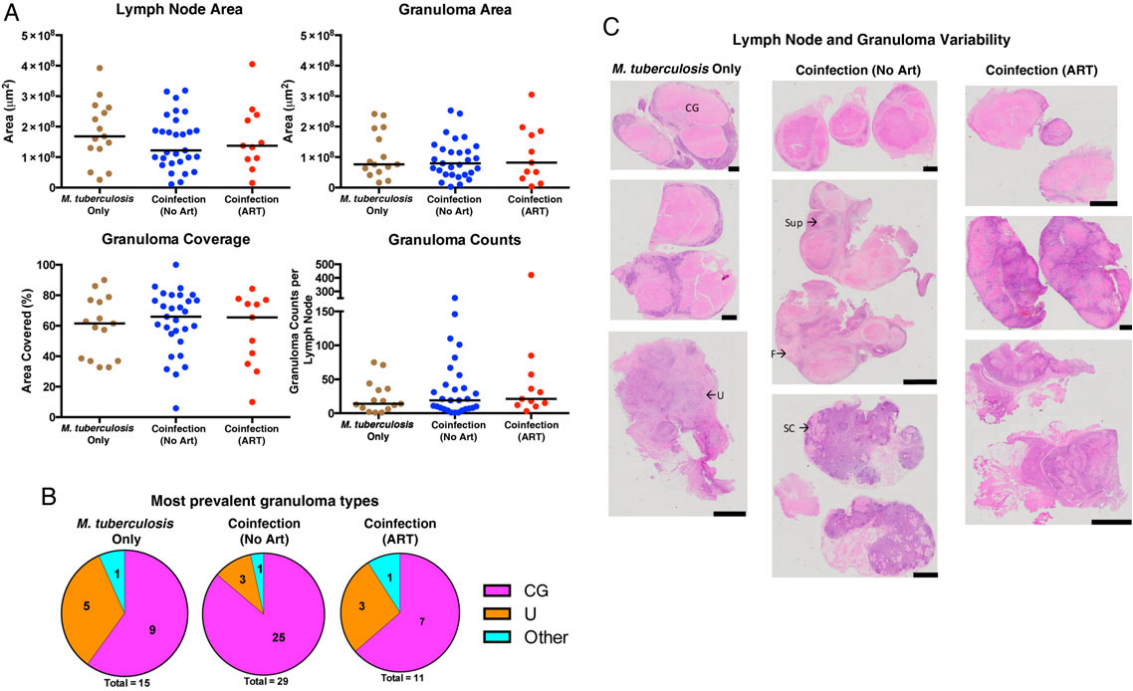
Persons coinfected with *Mycobacterium tuberculosis* and human immunodeficiency virus type 1 (HIV) do not have differences in lymph node size or granuloma area, counts, and type, compared with *M. tuberculosis*–monoinfected persons. *A*, No significant differences were observed in total cross-sectional areas of the excised lymph nodes and granulomas, percentage of lymph nodes covered in granulomas, or granuloma counts between *M. tuberculosis*–monoinfected persons and individuals coinfected with *M. tuberculosis* and HIV (who were or were not receiving antiretroviral therapy [ART]). *B*, The percentage of lymph nodes that contained the largest granulomas or unorganized inflammation within each infection group are displayed in each pie chart. *C*, Hematoxylin-eosin–stained slides demonstrating the heterogeneous nature of excised lymph nodes and their granulomas (black scale bar, 2 mm). A few granulomas and inflamed areas are labeled to demonstrate the high variability in granuloma types within individual lymph nodes. “Other” denotes suppurative (Sup), solid cellular (SC), and fibrotic (F) granulomas. CG, caseous granuloma; U, unorganized inflammation.

Biopsied LNs from persons with *M. tuberculosis* or HIV monoinfection or with coinfection, with or without ART, were analyzed for differences in pathology. Excised LNs from coinfected persons were not significantly different in cross-sectional area and did not contain differences in granuloma area, coverage, or counts (Figure 1*A*). When the type of granuloma was analyzed (necrotic, nonnecrotic, or unorganized inflammation), there were no significant differences between groups (*P* = .327 by χ^2^ analysis; Figure 1*B*). The extensive infiltration of these LNs with granulomas (Figure 1*C*) resulted in distortion of the normal LN architecture in most sections; as a result, it was not possible to determine whether HIV coinfection changes the normal features of LNs (such as germinal centers, interstitial space, and capsule).

### Coinfection Increases Bacterial Load Within LNs

**Figure 2.**
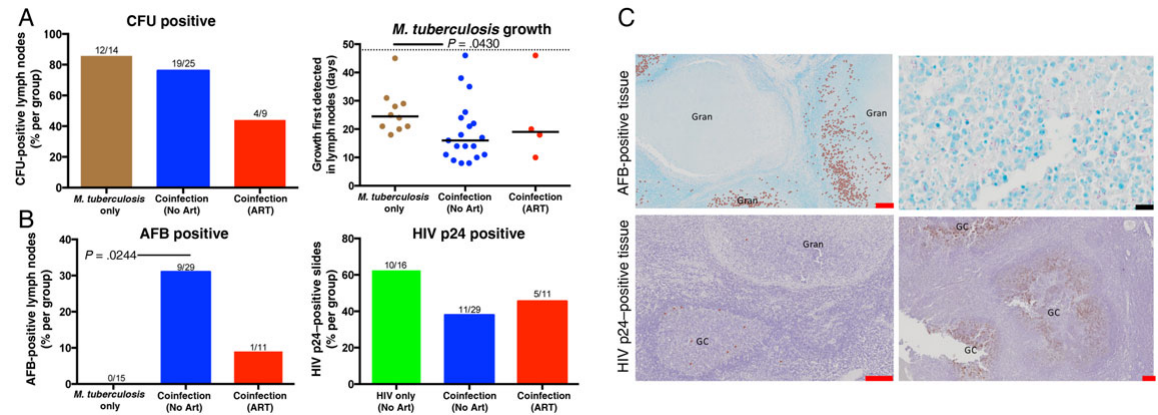
Lymph nodes from persons coinfected with *Mycobacterium tuberculosis* and human immunodeficiency virus type 1 (HIV) were more likely to contain higher bacterial loads, compared with *M. tuberculosis*–monoinfected persons. *A*, No difference in lymph node *M. tuberculosis* culture findings (ie, colony-forming units [CFU]) were observed among the 3 groups. Fractions above bars indicate positive lymph nodes over total lymph nodes (not all lymph nodes were cultured). Days to culture positivity (determined by the BACTEC MGIT system) was lowest within lymph nodes isolated from the coinfected recipients who were not receiving antiretroviral therapy (ART). The dotted line represents the culture-negative time limit (49 days). *B*, HIV was associated with an increased presence of acid-fast bacilli (AFB) that was not significantly reduced in the presence of ART. No AFB were observed in lymph nodes from HIV-uninfected persons. *M. tuberculosis* did not change the prevalence of HIV p24 among lymph nodes. *C*, AFB and HIV p24 staining within lymph nodes. AFB and HIV p24 are flagged in left panels. Significance was determined by the Kruskal–Wallis test with the Dunn multiple comparisons post hoc test (*P* = .05). Red and black scale bars represent 200 µM and 2 µM, respectively. GC, germinal center; Gran, granuloma.

When excised LNs were cultured for *M. tuberculosis*, no difference was observed between the groups in the rate of CFU positivity (Figure 2*A*). However, *M. tuberculosis* growth was observed after fewer days of culture in LNs from coinfected persons without ART, suggesting that those tissues had a higher bacterial burden than their counterparts [23]. Ziehl-Neelsen staining, which appeared to require a higher threshold of bacilli to be observed than CFUs, was also performed to identify *M. tuberculosis* presence. If at least 1 bacillus was located within the entire LN section, the sample was considered AFB positive. A greater proportion of LNs from coinfected persons without ART contained AFB (30% [9 of 29]), compared with LNs excised from *M. tuberculosis*–monoinfected persons (0% [0 of 15]) and coinfected persons receiving ART (9% [1 of 11]; Figure 2*B*). LNs from coinfected persons were just as likely to contain HIV p24 (11 of 29), irrespective of ART status (5 of 11; Figure 2*A*). One person receiving ART had an HIV p24–positive LN, despite having suppressed plasma viral loads. These data suggest that HIV increases the bacterial load within granulomas.

To determine where *M. tuberculosis* bacilli and HIV localize within infected LNs, we correlated AFB and HIV p24 presence with germinal centers or granulomas. Contrary to multiple models of granuloma formation [24, 25], *M. tuberculosis* primarily resided within the macrophage layer of granulomas and not within the caseum (Figure 2*C*). HIV p24 preferentially localized within germinal centers and not granulomas (Figure 2*C*), suggesting that minimal HIV replication was occurring within the granulomas. A qualitative observation suggests that the effect of HIV and *M. tuberculosis* on each other appear to be remote and not local because only 4 LN sections stained positive for both AFB and HIV p24.

### Coinfection Reduced Both CD4^+^ T-Cell Counts and CD4^+^/CD8^+^ T-Cell Count Ratios Within Excised Tissue That Were Partially Ameliorated by ART

**Figure 3.**
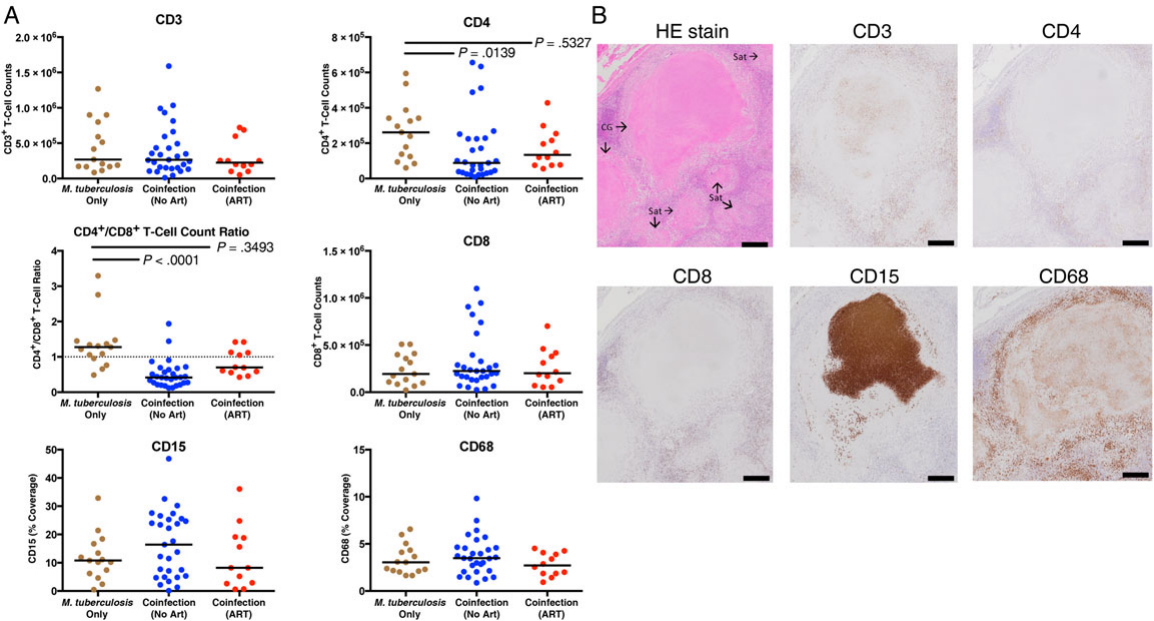
Antiretroviral therapy (ART) partially restores the reduction of CD4^+^ T cells and CD4^+^/CD8^+^ T-cell count ratio observed within tuberculosis-diseased lymph nodes from persons coinfected with *Mycobacterium tuberculosis* and human immunodeficiency virus type 1 (HIV). *A*, HIV reduces CD4^+^ T-cell counts and CD4^+^/CD8^+^ T-cell count ratios, compared with *M. tuberculosis*–monoinfected persons. No significant reduction was observed in coinfected patients receiving ART. The Kruskal–Wallis test and Dunn multiple comparisons post hoc test were used to determine significance. Adjusted *P* values are displayed. *B*, Hematoxylin-eosin (HE) or cellular staining in consecutive lymph node sections that contain caseous granulomas (CG) with satellite solid cellular granulomas (Sat; black scale bar, 100 µm). CD3, CD4, and CD8 staining predominately occurred within lymphocyte layer of granulomas. Interestingly, circular CD3 staining occurred occasionally, within the center of caseous granulomas without associated nuclei. CD15 was most often localized within caseous centers, without nuclei, and sporadically within macrophage layer of granulomas. CD68 staining was within caseous granulomas without associating with nuclei and was most frequently present in the macrophage layer of granulomas.

To determine the effect of coinfection on the cellular response within granulomas, we next investigated the numbers of CD3^+^, CD4^+^, and CD8^+^ T cells, monocytes (CD68 coverage), and neutrophils (CD15 coverage). Granulomas from coinfected persons contained significantly fewer CD4^+^ T cells, as well as a reduced CD4^+^/CD8^+^ T-cell count ratio, compared with HIV–uninfected persons (Figure 3*A*). This effect of low CD4^+^ T-cell count reduction in the group of samples from coinfected patients was partially restored in the group of ART recipients (Figure 3*A*). Moreover, coinfection did not affect CD3^+^ or CD8^+^ T-cell counts or CD15 and CD68 coverage within LNs. Although peripheral CD4^+^ T-cell counts were significantly lower (*P* = .03) they were, albeit weakly (*R*^2^ = 0.1515), correlated with granuloma CD4^+^ T-cell counts in ART-naive persons. This correlation was not significant when considering coinfected persons receiving ART (*R*^2^ = 0.08, *P* = .5328).

### Cellular and Cytokine Organization Within Granulomas

To further investigate the effect of coinfection on the cellular response in LNs, we next investigated the distribution of the cell types and their association with the cytokines IFN-γ, IFN-α, TNF, and IL-10. Neutrophil (CD15) staining was predominant within the center of caseous granulomas (Figure 3*B*). Macrophage (CD68) staining was also prevalent within caseous granulomas but frequently appeared in a pattern surrounding dense neutrophil staining (Figure 3*B*). CD3^+^, CD8^+^, and CD4^+^ T cells were evenly distributed within the lymphocyte layer of the granulomas in each group.

**Figure 4.**
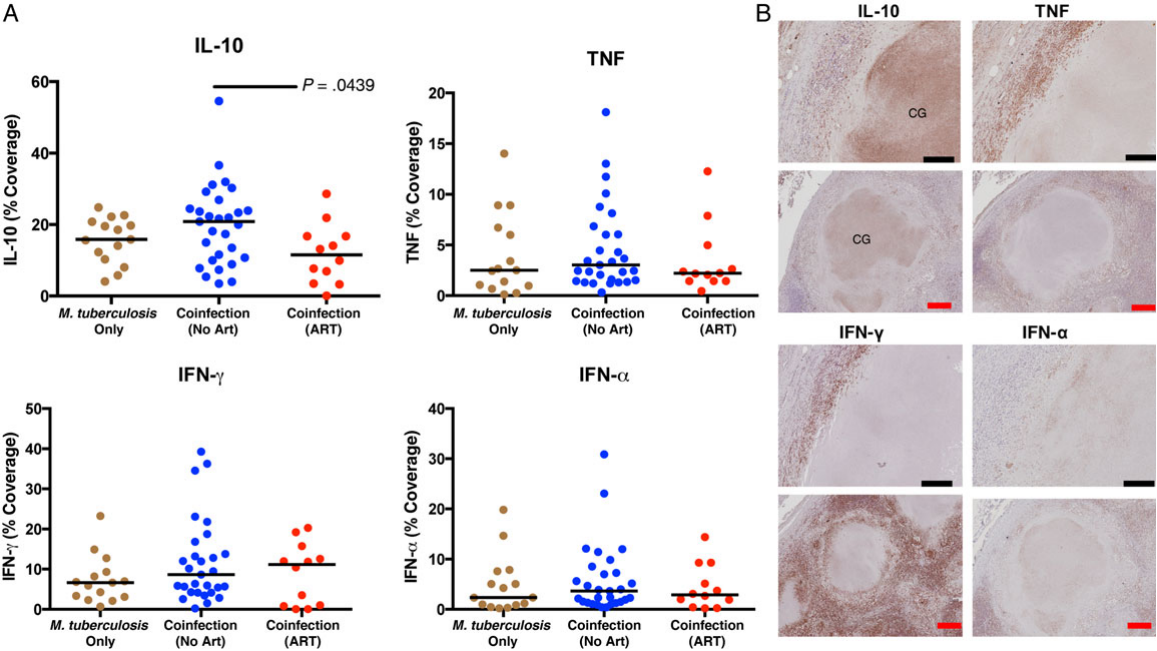
Antiretroviral therapy (ART) reduces interleukin 10 (IL-10) presence within tuberculosis-diseased lymph nodes. *A*, Percentage of lymph node sections covered by cytokines are represented. Human immunodeficiency virus type 1 (HIV) coinfection did not change IL-10 presence in lymph nodes, compared with *Mycobacterium tuberculosis* monoinfection. ART significantly reduced IL-10 presence in persons coinfected with *M. tuberculosis* and HIV. HIV did not change tumor necrosis factor (TNF), interferon γ (IFN-γ), or interferon α (IFN-α) presence in these tissues. The Kruskal–Wallis test and Dunn multiple comparisons post hoc test were used to determine significance. Adjusted *P* values are displayed. *B*, Cytokine staining in consecutive lymph node sections that contain caseous granulomas (CGs; black scale bar, 20 µm; red scale bar, 100 µM). Staining revealed that IL-10 was most often present in the center of caseous granulomas. TNF and IFN-γ were predominately identified in macrophage and lymphocyte layer. IFN-α most often localized within macrophage layer.

IFN-γ and TNF preferentially localized within the macrophage layer within granulomas. Multinucleated giant cells were more likely to contain IFN-α or TNF than IFN-γ or IL-10. HIV coinfection did not change IL-10, IFN-α, TNF, or IFN-γ presence within excised LNs (Figure 4*A*). However, ART status significantly reduced the IL-10 presence within LNs, compared with ART-naive coinfected persons (Figure 4*A*). IL-10 had the highest median percentage coverage of all cytokines investigated (Figure 4*A*) and was most often localized within caseous granulomas (Figure 4*B*). Total IL-10 coverage directly correlated with neutrophil influx within caseous granulomas, with HIV coinfection associated with a small reduction in this correlation (*M. tuberculosis*–monoinfected persons, *R*^2^ = 0.6936, *P* = .0001; coinfected persons, *R*^2^ = 0.3906, *P* = .0004).

### In Advanced HIV Infection, Granulomas Contain Fewer CD4^+^ and CD3^+^ T Cells, Lower CD4^+^/CD8^+^ T-Cell Count Ratios, and Increased Neutrophil, IL-10, and *M. tuberculosis* Presence

**Figure 5.**
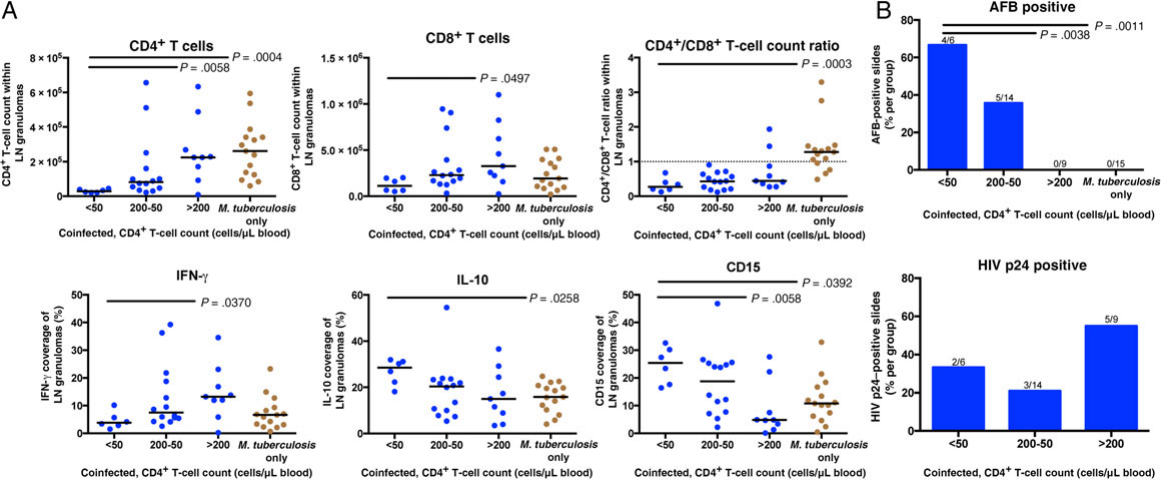
As human immunodeficiency virus type 1 (HIV) infection progresses, tuberculosis-diseased tissue contain fewer CD4^+^ T cells and lower CD4^+^/CD8^+^ T-cell count ratios, along with increased acid-fast bacilli (AFB), neutrophil, and IL-10 presence. *A*, Lymph nodes from persons who were coinfected with *M. tuberculosis* and HIV, not receiving ART, and <50 peripheral CD4^+^ T cells/µL of blood contain fewer CD4^+^ T cells, lower CD4^+^/CD8^+^ T-cell count ratios, and more IL-10 and CD15 than lymph nodes from *M. tuberculosis*–monoinfected persons. Lymph nodes from persons with <50 peripheral CD4^+^ T cells contain fewer CD4^+^ and CD8^+^ T cells along with less interferon γ (IFN-γ), compared with lymph nodes from coinfected persons with >200 peripheral CD4^+^ T cells/µL of blood. The Kruskal–Wallis test and Dunn multiple comparisons post hoc test were used to determine the significance of differences from persons with <50 peripheral CD4^+^ T cells/μL of blood. *B*, Lymph nodes from coinfected persons who were not receiving ART and had fewer peripheral CD4^+^ T cells were more likely to be positive for AFB than coinfected patients with >200 peripheral CD4^+^ T cells/µL of blood and those with *M. tuberculosis* monoinfection. Fractions above bars indicate positive lymph nodes over total lymph nodes. Significance was determined by Kruskal–Wallis test with the Dunn multiple comparisons post hoc test (*P* = .05). Adjusted *P* values are displayed.

To determine how granulomas changed as HIV immunodeficiency advanced, tissue from patients with varying peripheral CD4^+^ T-cell counts (>200, 200–50, <50 cells/µL of blood) were examined. Granuloma composition and bacilli presence varied significantly (Figure 5). Coinfected persons with <50 peripheral CD4^+^ T cells/µL of blood showed significantly lower granuloma CD4^+^ T-cell counts than in *M. tuberculosis*–monoinfected persons or those with >200 peripheral CD4^+^ T cells/µL of blood (Figure 5*A*). Severely depleted peripheral CD4^+^ T-cell counts also correlated with reduced CD4^+^/CD8^+^ T-cell count ratios, compared with the *M. tuberculosis*–monoinfected group (Figure 5*A*). Granulomas from coinfected persons with <50 peripheral CD4^+^ T cells/µL of blood contained less IFN-γ and CD8^+^ T cells than coinfected persons with >200 peripheral CD4^+^ T cells/µL of blood (Figure 5*A*). LNs from persons with <50 peripheral CD4^+^ T cells/µL of blood contained significantly more CD15 and IL-10 than *M. tuberculosis*–monoinfected and coinfected persons with >200 peripheral CD4^+^ T cells/µL of blood. As peripheral CD4^+^ T cell counts decreased, *M. tuberculosis* presence within granulomas increased (Figure 5*B*). Granulomas from persons with >200 peripheral CD4^+^ T cells/µL of blood were more likely to contain HIV p24 than persons with more-depleted peripheral CD4^+^ T cell counts (Figure 5*B*).

### *M. tuberculosis* Presence Correlates With Fewer CD4^+^ and CD8^+^ T Cells and More IL-10 and IFN-α

**Figure 6.**
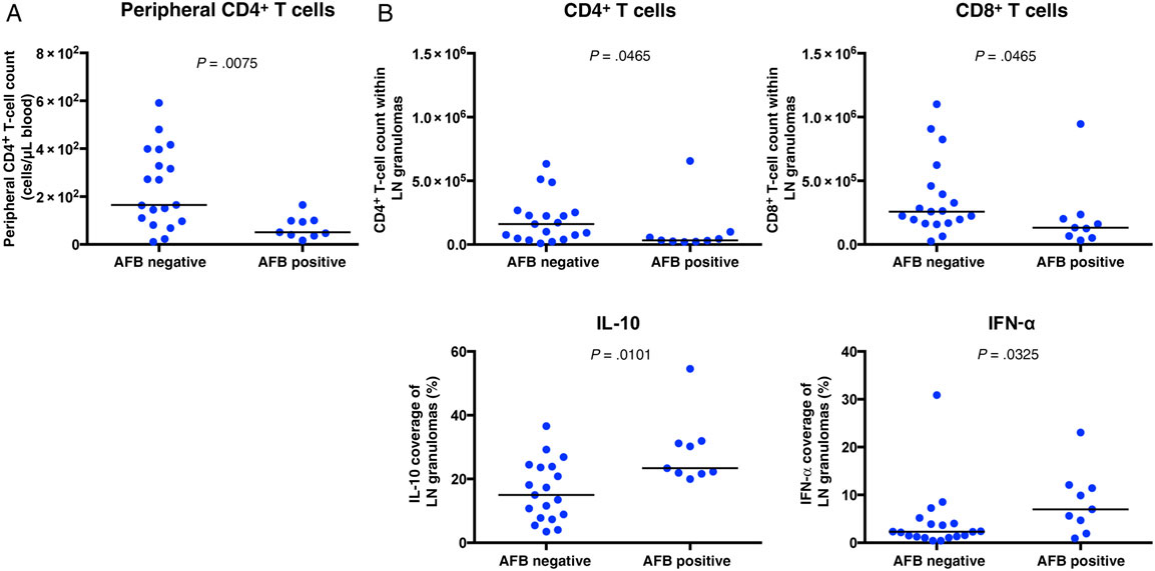
Acid-fast bacilli (AFB)–positive granulomas contained fewer CD4^+^ and CD8^+^ T cells along with increased interleukin 10 (IL-10) and interferon α (IFN-α) levels, compared with AFB-negative granulomas. *A*, Peripheral CD4^+^ T-cell counts were lower in coinfected persons with AFB-positive lymph node sections, compared with those with AFB-negative sections. *B*, AFB-positive lymph node sections contained fewer CD4^+^ and CD8^+^ T cells along with more IL-10 and IFN-α. No difference was observed in the expression of CD3, CD15, CD68, IFN-γ, or tumor necrosis factor (TNF) between AFB-positive and AFB-negative granulomas. The Mann–Whitney test was used to determine significance (*P* = .05).

To identify correlates of *M. tuberculosis* presence within granulomas, we quantified cellular and cytokine presence in AFB-positive and AFB-negative sections. AFB-positive sections were more likely to occur within persons with depleted peripheral CD4^+^ T cell counts (Figure 6*A*). AFB-positive tissue contained fewer CD4+ and CD8+ T cells than AFB- tissue (Figure 6*B*) and were more likely to contain more IL-10 and IFN-α (Figure 6*B*).

## DISCUSSION

If disruption within granulomas leads to *M. tuberculosis* reactivation [26] and if increases in bacterial growth within granulomas is associated with active tuberculosis [14, 27], it is likely that the ability of HIV to increase *M. tuberculosis* presence within granulomas may lead to increased tuberculosis pathology. To this end, we examined the microenvironment of granulomas from coinfected and *M. tuberculosis*–monoinfected persons to determine how HIV increases *M. tuberculosis* presence within granulomas.

*M. tuberculosis* growth restriction is the primary objective of granuloma function, so it was striking that the only observed AFB-positive LN sections and the higher bacterial burdens were from coinfected persons. Since there was no difference observed in cultured *M. tuberculosis* from LNs of each group but a higher bacterial load, HIV might not increase *M. tuberculosis* presence, but it does increase *M. tuberculosis* growth. Similarly, granulomas from coinfected persons tend to contain more bacilli than those from HIV–uninfected persons [28–30]. These granulomas were also more likely to contain fewer CD4^+^ T cells and lower CD4^+^/CD8^+^ T-cell count ratios. This increase in AFB positivity indirectly correlated with peripheral CD4^+^ T-cell count and the depletion of granulomatous CD4^+^ T cells, demonstrating that, as HIV infection clinically progresses, the likelihood of *M. tuberculosis* growth also increases [28, 30]. The ability of ART to ameliorate the loss of CD4^+^ T cells within granulomas may be why this therapy reduced tuberculosis incidence across persons with a wide spectrum of peripheral CD4^+^ T-cell counts [31].

As HIV infection advances, granulomas undergo phenotypic change. Within persons with <50 peripheral CD4^+^ T cells/µL of blood, a significant reduction in granulomatous CD4^+^ and CD8^+^ T cells, reduced IFN-γ production, and increased *M. tuberculosis* abundance were observed. The reduction of CD4^+^ and CD8^+^ T cells and IFN-γ production within granulomas may directly lead to increased *M. tuberculosis* abundance because of their well-established role in killing *M. tuberculosis* (reviewed in [32]).

CD4^+^ T cells have been identified as the primary site for HIV replication within granulomas [33]. It is hypothesized that HIV replication readily occurs within granulomas, owing to their high composition of activated CD4^+^ T cells and macrophages [22, 34]. However, HIV presence within LNs occurred more often within germinal centers than in granulomas, which may have resulted from a higher concentration of HIV–infectable cells. This suggests that granulomas may not be ideal sites for HIV replication.

The significant neutrophil debris staining within caseum, especially within peripheral CD4^+^ T-cell–depleted persons, suggests that neutrophils are actively migrating into granulomas. Neutrophil presence within granulomas increases inflammation and is associated with active tuberculosis [35, 36]. We hypothesize that this influx of neutrophils (a proinflammatory response) in LNs of patients with <50 peripheral CD4^+^ T cells/μL of blood ultimately led to the increased production of IL-10 (an antiinflammatory response), since their presence was positively correlated within caseous granulomas. The increase in *M. tuberculosis* numbers that was associated with IL-10 may also result from its ability to inhibit IFN-γ–induced macrophage killing of *M. tuberculosis* [37], and it can reduce *M. tuberculosis*–specific T-cell proliferation in humans [38].

Similar to IL-10, IFN-α was more likely to be present in AFB-positive granulomas. Because IL-10 can inhibit IFN-α production [39], and IFN-α can induce IL-10 production to reduce the ability to restrict *M. tuberculosis* growth [40], we hypothesize that the presence of IFN-α and IL-10 within granulomas may lead to an increase in *M. tuberculosis* growth [14].

**Figure 7.**
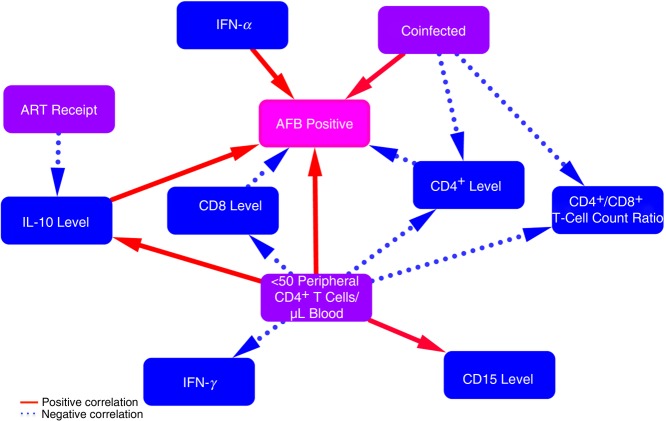
Proposed network of how human immunodeficiency virus type 1 (HIV) and treatment status change phenotypic interactions within *Mycobacterium tuberculosis* granulomas. Red solid and blue dotted lines represent positive and negative correlations, respectively, between phenotypic changes within granulomas (blue and pink boxes) and clinical descriptions of persons (purple boxes). Arrows represent our hypotheses as to the cause of the phenotypic changes. Coinfection increases acid-fast bacilli (AFB) presence and reduces CD4^+^ T-cell counts within granulomas. As HIV infection progresses (to <50 peripheral CD4^+^ T cells/μL of blood), granulomas contain less CD4, CD8, and interferon γ (IFN-γ) and more CD15 and interleukin 10 (IL-10), which lead to increased *M. tuberculosis* growth. IL-10 and IFN-α lead to more *M. tuberculosis* growth within granulomas. Antiretroviral therapy (ART) receipt reduces IL-10 presence.

To understand what was occurring within the granulomas, we elucidated a correlation network of multiple phenotypic changes within these granulomas (Figure 7). Although a limitation of immunohistochemistry is the inability to differentiate cause and effect, it provides an opportunity to develop these hypotheses and build support with in vitro and ex vivo evidence. We hypothesize that HIV directly kills CD4^+^ T cells within granulomas and that this worsens as HIV disease progresses. Initially, HIV replication increases prior to severe peripheral CD4^+^ T-cell loss, which leads to an increase in CD8^+^ T-cell counts and IFN-γ production. As peripheral CD4^+^ T cells continue to deplete, neutrophil recruitment intensifies, followed by the production of IL-10 and IFN-α, which leads to further suppression of T-cell function [41] and *M. tuberculosis* growth. The ability of ART to restore the CD4^+^ T-cell count and reduce IL-10 production within granulomas of coinfected persons may indirectly lead to reduced *M. tuberculosis* growth. Furthering our understanding of how HIV and ART change *M. tuberculosis* granulomas will help elucidate the unknown factors that HIV exploits to increase *M. tuberculosis* susceptibility and tuberculosis pathology.
